# Supplementary material for: The c-Jun N-terminal kinase pathway of a vector insect is activated by virus capsid protein and promotes viral replication
Source: eLife. 2017 Jul 18;6:e26591. doi: 10.7554/eLife.26591 (PMC5515582; doi:10.7554/eLife.26591)
Supplement: Supplementary file 1. — DOI: http://dx.doi.org/10.7554/eLife.26591.029 [file elife-26591-supp1.docx]

**Supplementary file 1.** **Primers used in this study**

| **Primer name** | **Primer sequence** |
| --- | --- |
| gps2-F | CAACTTTCATGTTTCGCTCG |
| gps2-R | GACCTCTCTCTGCATGTTGGAG |
| cp-F | ATGGGTACCAACAAGCCAGC |
| cp-R | CTAGTCATCTGCACCTTCTG |
| ubc13-F | ATGGCAGCCTTACCACGGAG |
| ubc13-R | TCAATTGTCCATGGCGTATC |
| ubc13-his-F | AGGAGATATACCATGGGCATGGCAGCCTTAC |
| ubc13-his-R | CCGCAAGCTTGTCGACATTGTCCATGGCG |
| gps2-his-F | GGAATTCCATATGATGCTAAAATCAGGAGGTGG |
| gps2-his-R | CGGGATCCTTAGACCTCTCTCTGCATG |
| gps2N-his-F | AGGAGATATACCATGGGCCTAAAATCAGGAGGTGGCCA |
| gps2N1-his-R | GCCGCAAGCTTGTCGACGGCGGGTAGGAGATTATGAG |
| gps2N2-his-R | GCCGCAAGCTTGTCGACACCGTACTGATTATTTTTGT |
| gps2N3-his-R | GCCGCAAGCTTGTCGACTGGATGTTTGACAGAGGGTG |
| gps2N4-gst-F | GGTCGTGGGATCCCCGGGTCTGGAGGCTCCTTGAAAAGG |
| gps2N4-gst-R | TCACGATGAATTCCCGGGTGGTGGCGGTTGGCTTTCTTG |
| gps2C-his-F | AGGAGATATACCATGGGCCCCTCTGTCAAACATCCAAC |
| gps2C-his-R | GCCGCAAGCTTGTCGACGACCTCTCTCTGCATGTTGG |
| cp-his-F | GGAATTCCATATGATGGGTACCAACAAGCCAGC |
| cp-his-R | CGGGATCCCTAGTCATCTGCACCTTCTG |
| cp-gst-F | CGGGATCCATGGGTACCAACAAGCCAGC |
| cp-gst-R | TCCCCCGGGCTAGTCATCTGCACCTTCTG |
| ubc13-gst-F | GGTCGTGGGATCCCCGGGATGGCAGCCTTAC |
| ubc13-gst-R | TCACGATGAATTCCCGGGATTGTCCATGGCG |
| jnk1-his-F | AGGAGATATACCATGGGCATGCGTCTGCTGACCTAC |
| jnk1-his-R | CCGCAAGCTTGTCGACCCGACGGGTGGTGGGGG |
| jnk2-his-F | AGGAGATATACCATGGGCGTAAACTCCTTTTTAAT |
| jnk2-his-R | CCGCAAGCTTGTCGACGGTGCGATTTGAATTCA |
| gps2-dsRNA-F | TAATACGACTCACTATAGGCATCCAACAACCTCCTCAG |
| gps2-dsRNA-R | TAATACGACTCACTATAGGGAGATAGCATAGCGTCCAG |
| jnk1-dsRNA-F | TAATACGACTCACTATAGGNCCTTGTCGCACTCAGAAT |
| jnk1-dsRNA-R | TAATACGACTCACTATAGGNCTCCAACCTCCACAGTATAG |
| jnk2-dsRNA-F | TAATACGACTCACTATAGGGATGTCTACCTCGTTATGGA |
| jnk2-dsRNA-R | TAATACGACTCACTATAGGGCAATGTTACTTGGCTTCA |
| ubc13-dsRNA-F | TAATACGACTCACTATAGGCCGTGCCTGGAATAAGTG |
| ubc13-dsRNA-R | TAATACGACTCACTATAGGGCCGACATTGGGTAATCTT |
| TNF-α-dsRNA-F | TAATACGACTCACTATAGGATGCTGACTGATGAAGATGA |
| TNF-α-dsRNA-R | TAATACGACTCACTATAGGGCTCTATCCGCCATTCTC |
| gfp-dsRNA-F | CACAAGTTCAGCGTGTCCG |
| gfp-dsRNA-R | GTTCACCTTGATGCCGTTC |
| cp-q-F | AGTGCTGATCGTATTGACAGA |
| cp-q-R | GATGAAGTACACAACTGGTC |
| gps2-q-F | ATGACAATAGAAGGCGACAA |
| gps2-q-R | TTATGAGGTGGCGGAACT |
| jnk1-q-F | CTCAGAGATCACTGGAAGAA |
| jnk1-q-R | TAGAGCAGATAGGACATTCG |
| jnk2-q-F | GGATGTATTATGGCTGAGATG |
| jnk2-q-R | AAGTCCTGAGATGGTGTTC |
| ubc13-q-F | CCAATCCAGACGATCCTCT |
| ubc13-q-R | GCGTATCTCCGAGTCCAT |
| TNF-α-q-F | GCACCACTTACACCAATCA |
| TNF-α-q-R | CGAACATAGAGGCTGTCTC |
| ef2-q-F | GTCTCCACGGATGGGCTTT |
| ef2-q-R | ATCTTGAATTTCTCGGCATACATTT |
